# Molecular Signature of the Ebola Virus Associated with the Fishermen Community Outbreak in Aberdeen, Sierra Leone, in February 2015

**DOI:** 10.1128/genomeA.01093-15

**Published:** 2015-09-24

**Authors:** Maria R. Capobianchi, Cesare E. M. Gruber, Fabrizio Carletti, Silvia Meschi, Concetta Castilletti, Francesco Vairo, Mirella Biava, Claudia Minosse, Gino Strada, Gina Portella, Rossella Miccio, Valeria Minardi, Luca Rolla, Abdul Kamara, Giovanni Chillemi, Alessandro Desideri, Antonino Di Caro, Giuseppe Ippolito

**Affiliations:** a National Institute for Infectious Diseases “L. Spallanzani,” Rome, Italy; b Emergency NGO, Milan, Italy; c National Laboratory Services, Ministry of Health and Sanitation, Freetown, Sierra Leone; d CINECA, Rome, Italy; e Department of Biology, University of Rome Tor Vergata, Rome, Italy; f Molecular Digital Diagnostics (MDD), Viterbo, Italy

## Abstract

We report the complete genome sequence of Ebola virus from a health worker linked to a cluster of cases occurring in the fishing community of Aberdeen, Sierra Leone (February 2015), which were characterized by unusually severe presentation. The sequence, clustering in the SL subclade 3.2.4, harbors mutations potentially relevant for pathogenesis.

## GENOME ANNOUNCEMENT

An unprecedented Ebola outbreak is still ongoing in West Africa (1, 2). Early reports suggest that Ebola virus (EBOV) is mutating faster than previously observed, with the potential for changes in its transmissibility and virulence (3, 4).

During the week of February 18, 2015, a cluster of cases were reported in the highly mobile fishing community of Aberdeen Seashores, Freetown, Sierra Leone (5) with a subsequent peak of new cases in the northern district of Bombali and in Freetown (6, 7). The Bombali outbreak was reportedly linked to the Freetown fishing community cluster, probably shuttled by a fisherman who got sick in Freetown and traveled to Bombali seeking care and help from his family (8). The patients from the cluster, attending the Emergency (NGO) Ebola Treatment Center in Goderich, Freetown (GETC), presented unusually severe symptoms (9), suggesting the implication of a more virulent strain.

We obtained the complete EBOV genome sequence (Ebola virus/*H. sapiens*-wt/SLE/2015/Makona-Goderich1) from a European GETC health care worker (HCW) who was infected while attending patients from the Aberdeen fishermen community. Viral RNA was extracted from plasma at admission on February 19, 2015. The complete genome was amplified in 45 overlapping fragments and Sanger sequenced (10).

In the maximum-likelihood phylogenetic tree, the new sequence clusters with the Sierra Leone subclade 3.2.4 described by Tong et al. (11). The linkage with the “fishermen outbreak” is supported by the almost complete identity (only 2 and 5 single nucleotide variants) with two contemporary partial sequences (10,940 and 11,133 nt) from two fishermen who attended GETC (not shown). Considering the presently available EBOV sequences, retrieved from NCBI (12) and Virogical.org (13), these three sequences are phylogenetically grouped in a significant cluster of contemporary sequences deriving mostly from Bombali, Western Rural, and Western Urban districts.

The viral sequence from the HCW shares with the two partial fishermen sequences three uncommon nonsynonymous substitutions, one in NP (c1958t:P497S) and two in GP (a7267t:R410S and a7352g:K439E). Among the Sierra Leone EBOV sequences available so far, these mutations are represented only in the mentioned phylogenetic cluster. Particularly, the NP mutation alone, located in a B-cell epitope (14, 15), is observed in five sequences from Bombali, two from Western Urban, two from Western Rural, one from Western Area, and one from an unknown district. Both GP mutations are located in the highly glycosylated mucin-like domain implicated in EBOV cell entry and immune evasion; the position 410 is part of a B-cell epitope that is a dominant target for humoral response (15–17). GP mutations alone are observed in 19 sequences from Bombali, two from Western Urban, one from Port Loko, and two from unknown districts; both NP and GP mutations are present in a subcluster of sequences that includes five contemporary sequences from Bombali and Western Urban districts (WTSI/UoC/PHE|1805_C1_MK2371|SLE|Bombali|2015-02-25, WTSI/UoC/PHE|1805_C1_MK2395|SLE|Bombali|2015-02-25, WTSI/UoC/PHE|1804_C1_MK2570|SLE|Bombali|2015-03-01, WTSI/UoC/PHE|1105_C1_MK2790|SLE|Bombali|2015-03-07, and WTSI/UoC/PHE|3004_C2_KT5214|SLE|WesternUrban|2015-02-20); and one from the United Kingdom (KR025228.1). These mutations may represent a molecular signature of the “fishermen outbreak” and may be pathogenetically relevant. Functional studies are necessary to address this point.

### Nucleotide sequence accession number.

The complete genome sequence of Ebola virus/*H. sapiens*-wt/SLE/2015/Makona-Goderich1 has been deposited in GenBank under the accession number KT345616.
